# A diet containing soybean oil heated for three hours increases adipose tissue weight but decreases body weight in C57BL/6 J mice

**DOI:** 10.1186/1476-511X-12-26

**Published:** 2013-03-06

**Authors:** Meera Penumetcha, Mary K Schneider, Holly A Cheek, Sonia Karabina

**Affiliations:** 1 Division of Nutrition, Byrdine F. Lewis School of Nursing and Health Professions, Georgia State University, Paris, Cedex 12, France; 2 INSERM UMRS 933, UPMC-Paris 6, 26, avenue Arnold Netter, 75571, Paris, Cedex 12, France

**Keywords:** Heated oil, Oxidized oil, Adipose mass, Diet

## Abstract

**Background:**

Our previous work showed that dietary oxidized linoleic acid given, as a single fatty acid, to LDL receptor knockout mice decreased weight gain as compared to control mice. Other studies have also reported that animals fed oils heated for 24 h or greater showed reduced weight gain. These observations, while important, have limited significance since fried foods in the typical human diet do not contain the extreme levels of oxidized lipids used in these studies. The main goal of this study was to investigate the effects of a diet containing soybean oil heated for 3 h on weight gain and fat pad mass in mice. Additionally, because PPARγ and UCP-1 mediate adipocyte differentiation and thermogenesis, respectively, the effect of this diet on these proteins was also examined.

**Findings:**

Four to six week old male C57BL/6 J mice were randomly divided into three groups and given either a low fat diet with heated soybean oil (HSO) or unheated soybean oil (USO) or pair fed for 16 weeks. Weight and food intake were monitored and fat pads were harvested upon the study’s termination. Mice consuming the HSO diet had significantly increased fat pad mass but gained less weight as compared to mice in the USO group despite a similar caloric intake and similar levels of PPARγ and UCP1.

**Conclusion:**

This is the first study to show that a diet containing soybean oil heated for a short time increases fat mass despite a decreased weight gain in C57BL/6 J mice. The subsequent metabolic consequences of this increased fat mass merits further investigation.

## Findings

### Background

Foods rich in polyunsaturated fatty acids (PUFA) are susceptible to oxidation. Food processing, storage and cooking at high temperatures hasten this process and result in the formation of both primary (peroxide value, PV; conjugated dienes, CD) and secondary products of lipid oxidation. Although studies that investigate the biological effects of these dietary oxidized lipids (DOL) in humans are limited, rodents that are fed diets containing large proportions (50-100% of total fat) of oils heated for prolonged periods (10 h to 38 d) gain less weight and show a reduction in fat mass as compared to controls [1-3]. A literature review of commonly consumed foods, however, demonstrates that French fries, fried fish, pizza and nuts have PVs of 1.98, 2.2, 2.45 and 1.20 meq/Kg, respectively [4], which are significantly lower than those used in previous animal studies. We thus believe that these studies do not accurately represent the effects of typical DOL consumption in the human population. From the above mentioned studies it appears that the weight reducing effects of DOL are probably due to the high amounts of oxidized lipids used. In order to address this issue, we used a diet containing oil heated for only 3 h, comprising about 1/3 of total dietary fat (wt/wt), and tested its effects on total body weight and fat pad mass. As consumption of fried and processed foods is increasing globally it is important to know how DOL influence adipose tissue mass and subsequent metabolic risk.

### Methods

#### Animals

Twenty-four, 6–8 week old, male C57BL/6 J mice were purchased from the Jackson Laboratory (Bar Harbor, ME). The Georgia State University (GSU) Animal Care Committee approved all protocols (protocol #A060654) based on the Helsinki Declaration and mice were treated in compliance with the GSU Animal Care Committee regulations. Mice were randomly divided into three groups. One group was fed, *ad libitum,* a low fat diet with one-third of the total fat provided by unheated soybean oil (USO) while a second group was fed *ad libitum* a low fat diet with one-third of the total fat provided by heated soybean oil (HSO). A third group of mice was pair-fed (PSO) to the HSO group. In other words, though the PSO mice were fed the diet containing unheated oil, they were only allowed to consume the same amount of food (in grams) as the HSO group. A known amount of fresh food was placed in the cage every two days and any remaining or spilled food was weighed and discarded. Mice were weighed once weekly on a digital scale. After 16 weeks, mice were euthanized following an overnight fast. An incision was made in the abdomen and epididymal white adipose tissue (EWAT) was removed. The mouse was then placed on its ventral side and the interscapular brown adipose tissue (IBAT) was carefully excised. Fat pads were quickly rinsed in ice cold PBS, weighed on a high precision balance, flash frozen in liquid nitrogen and stored at −80°C. Methods for Western blot and RT-QPCR are reported in Additional file 1.

#### Mouse diet

HSO diet was produced by heating soybean oil on a hot plate at 190°C for 3 hours with an air compressor providing a continuous flow of oxygen throughout the oil. Both heated and unheated soybean oils were sent to Research Diets Inc. (New Brunswick, NJ) on dry ice to be incorporated into pelleted mouse chow. Each diet was distinctively colored to facilitate observation of fecal samples. Mouse diet was stored at 4°C to prevent further oxidation.

PV of the mouse chow was determined by CII Laboratory Services (Kansas City, MO). Lipids were extracted from all diets using a solvent system of hexane and isopropanol (HIP) (3:2) as described by Radin [5]. Conjugated dienes and TBARS (secondary product of oxidation) of the extracted lipids were determined as described previously [6]. Compositions of the diets along with the values for products of oxidation are listed in Additional file 2: Table S2.

### Statistical analysis

Statistics were generated using SPSS version 16.0 (SPSS Inc., Chicago, IL) using either one way-ANOVA followed by Bonferroni Post hoc tests or Kruskal-Wallis test followed by Mann Whitney test depending on the distribution. Statistical significance was set at P≤0.05.

### Results

**Figure 1:**
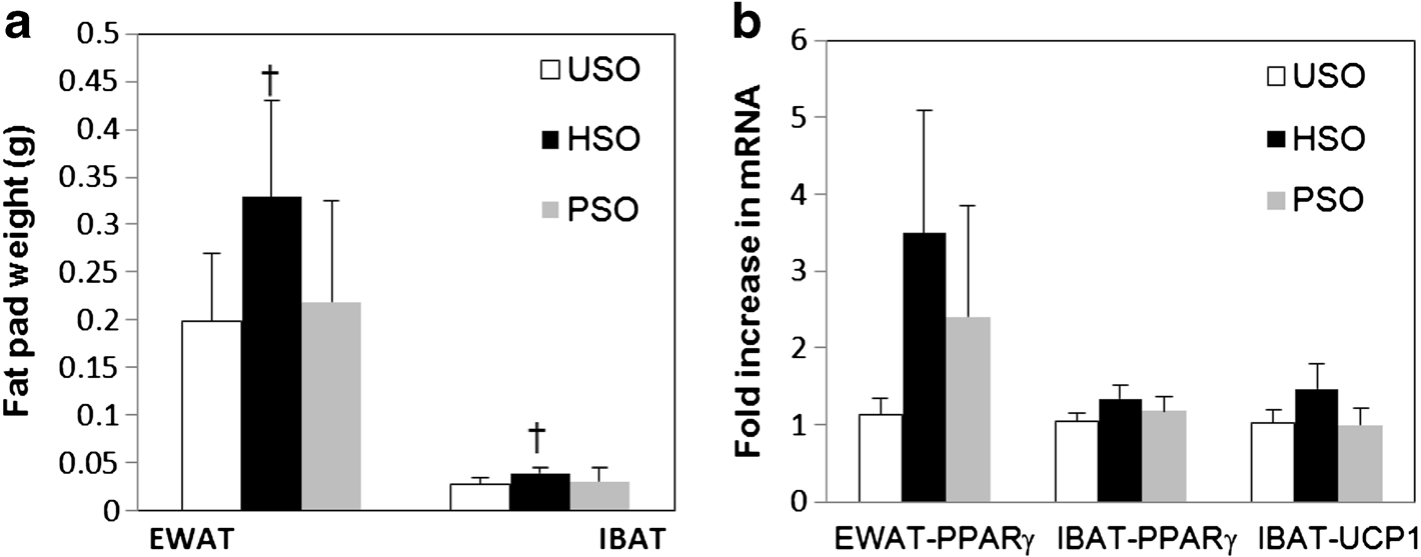
**Mean fat pad weights (1a) and mRNA expression (1b) of mice fed a USO (white bar; n=8), HSO (black bar; n=8) or PSO (grey bar; n=8) diet.** Bars represent mean ± SD. Group means were compared by the Kruskal Wallis test and pair wise comparisons were made by Mann Whitney U test. Statistical significance was set at p≤0.05. Means with “†” are significantly different as compared to USO and PSO groups.

Food intake among the USO, HSO and PSO groups was not different (Table 1). However, mice fed the USO diet gained the most weight in comparison to mice that were pair fed or consumed the HSO diet (Table 1) Mean body weights over time are shown in Additional file 3: Figure S2. Food efficiency was similar in the USO and HSO groups but was significantly lower in the PSO group (Table 1). Despite the lesser weight gain in the HSO group as compared to the USO group, EWAT and IBAT weights were significantly higher in the HSO group compared to the USO and PSO groups (Figure 1a). EWAT PPARγ mRNA was not different between the groups (Figure 1b). PPARγ and UCP1 mRNA levels in IBAT were not different between the three groups (Figure 1b). PPARγ and UCP-1 protein expression levels were not different between the groups in both EWAT and IBAT (Table 1).

**Table 1:** Body weight gain, food intake and other parameters (mean±SD) in mice fed a USO, HSO or PSO diet

**Parameter**	**USO**	**HSO**	**PSO**
Food intake (g/wk)	20.65 ± 0.09^a^	18.4 ± 0.05^a^	18.38 ± 0.19^a^
Weight gain (g)	8.86 ± 1.37^a^	7.10 ± 1.47^b^	5.71 ± 1.13^c^
Food efficiency (weight gain, g/food intake, g)	0.031 ± 0.005^a^	0.028 ± 0.006^ab^	0.022 ± 0.004^b^
EWAT PPARγ protein (PPARγ/GAPDH)	0.226 ± 0.08^a^	0.264 ± 0.12^a^	0.456 ± 0.6^a^
IBAT UCP-1 protein (UCP-1/Sypro protein stain)	0.139 ± 0.16^a^	0.188 ± 0.16^a^	0.127 ± 0.20^a^

*USO*=Unheated soybean oil diet; *HSO*=Heated soybean oil diet; *PSO*=Pair-fed to HSO with USO. Differences between the three groups were compared with one-way ANOVA followed by Bonferroni pair wise comparisons. Mean values within a row, with different superscripts of the alphabet, are significantly different from one another. N=8 per group for all the parameters except for food intake. Mice were housed at 2-3/cage and so n=3 for food intake (p≤0.05).

### Discussion

We successfully created a mouse diet that contained mostly primary products of oxidation (PV and CD) and negligible amounts of secondary products of oxidation (TBARS). In this study, mice that consumed a diet containing 1/3 of the total fat from heated oil gained less weight as compared to mice that consumed the unheated oil diet. This decrease in weight gain was 6% in our study as compared to 15**-**55% in other studies [1-3,7]. In those studies, oils were highly oxidized and contained 150 times the PV and 1500 times the TBARS as compared to the diet in this study. Oxidized oils have been shown to decrease fat absorption [8] and increase fecal fat excretion. Feces from mice across all groups looked similar and were *not* oily. Thus, the lower weight gain in the HSO group could be due to factors other than decreased fat absorption. Surprisingly, pair-fed mice (PSO) gained less weight than the HSO group, despite a similar caloric intake and physical activity (data not shown). Pair feeding, however, required that only a small amount of food (3–4 pellets) be placed in the feeding hopper and this might have forced the mice to exert more energy to access the food, resulting in increased energy expenditure.

A novel finding in our study is the increase in fat pad mass seen in mice consuming mildly heated soybean oil as compared to mice in the USO and PSO groups. Studies by Chao et al., in mice [1] and by others in rats [9] have shown that diets containing heated oils decrease fat pad mass and body weight, respectively. As mentioned before, this could be reflective of the deficit in calories due to decreased fat absorption. The gain in fat mass in this study cannot be explained by increased food intake because there were no differences in food intake between the USO and HSO groups. One explanation for the increased fat pad weight among HSO as compared to USO mice may be the repartitioning of fat and lean tissue. For example, conjugated linoleic acid, which is very similar in structure to oxidized PUFA (hydroxy linoleic acid), has been shown to change the proportions of lean and fat mass in the absence of weight change [10]. In addition, primary products of lipid oxidation, but not secondary products, have been shown to act as ligands of PPARγ [11]. Activation of PPARγ promotes modest increases in fat mass as seen in mice [12] and humans [13] treated with rosiglitazone, a known PPARγ agonist. We hypothesized that the increase in fat pad mass among mice consuming HSO could be due to an upregulation of PPARγ. Both mRNA and protein levels of PPARγ, however, were not different in the EWAT and IBAT between the groups. This suggests that mildly heated soybean oil might not change PPARγ mRNA or protein levels, but it cannot be ruled out that it could activate PPARγ. Furthermore the large variance in measuring PPARγ along with the small sample size might have made it difficult to find a statistically significant difference.

A limitation of this study is that energy expenditure was not measured in these mice and would have answered the question of whether oxidized lipids could alter basal metabolic rate thereby explaining the differences in weight gain. Thermogenesis is a component of energy balance. We wanted to see if the reduced weight gain in the HSO group could be attributed to an energy deficit and measured IBAT UCP-1, a recognized cellular marker of thermogenesis. As we report here, there were no differences in the UCP-1 mRNA or protein levels between any of the groups. The lack of difference in UCP-1 levels in the present study, however, does not negate a role for thermogenesis. Evidence from studies in mice show that changes in thermogenesis can occur independent of UCP-1 expression [14]. In addition, measuring thermogenesis by implanting a subcutaneous sensor would yield a direct measurement.

In conclusion, this is the first study to show that dietary oxidized lipids, in amounts found in commonly consumed foods, can increase fat pad mass. The metabolic consequences of this increased fat mass with regard to glucose homeostasis merits further investigation.

## Abbreviations

HSO: Heated soybean oil; PSO: Mice pair-fed with unheated soybean oil; EWAT: Epididymal white adipose tissue; IBAT: Interscapular brown adipose tissue; PPARγ: Peroxisome proliferator activated receptor gamma; UCP1: Uncoupling protein 1; DOL: Dietary oxidized lipids; CD: Conjugated diene; PV: Peroxide value

## Competing interests

The authors declare that they have no competing interests to report.

## Authors’ contributions

MP designed the study, participated in animal work, analyzed and interpreted the data and drafted the manuscript. MKS participated in the weighing, feeding and surgical procedures of mice, Western blot analysis of PPARγ and reviewed the manuscript. HAC carried out the Western blot analysis of UCP-1 and reviewed the manuscript. SK was responsible for carrying out the RT-QPCR, data analysis and reviewed the manuscript. All authors read and approved the final manuscript.

## Supplementary Material

Additional file 1: Methods Western blots and RT-QPCR. Click here for file

Additional file 2: Table S2 Composition of the HSO^a^ and USO^b^ diets. Click here for file

Additional file 3: Figure S2 Mean body weights of mice over the 16 week study period. Click here for file
