# Supplementary material for: A diet containing soybean oil heated for three hours increases adipose tissue weight but decreases body weight in C57BL/6 J mice
Source: Lipids Health Dis. 2013 Mar 6;12:26. doi: 10.1186/1476-511X-12-26 (PMC3599973; doi:10.1186/1476-511X-12-26)
Supplement: Additional file 1: Methods — Western blots and RT-QPCR. [file 1476-511X-12-26-S1.doc]

**Additional File 1:**

**Methods:**

Cytosolic and nuclear fractions were extracted from the EWAT and IBAT by the method of Freedman, SD and stored at -80^o^C until further analysis. Protein concentrations of the cytosolic and nuclear extracts were determined using a bicinchoninic acid assay kit (Sigma, St. Louis, MO). Absorbance was read at 562 nm.

**Western for PPARγ:** Approximately 50 µg of the cytosolic protein extract from EWAT was separated by 10 % SDS-PAGE. The gel was transblotted onto a polyvinylidene fluoride (PVDF) membrane which was then immunoblotted for PPARγ (1:400 rabbit polyclonal primary antibody, then 1:4000 goat anti-rabbit IgG HRP secondary antibody). The same membrane was then immunoblotted for GAPDH (1:1000 mouse monoclonal anti-rabbit primary antibody followed by 1:4000 goat anti-mouse IgG HRP secondary antibody). Signals were detected using a chemiluminescence kit (Immobilon Western Chemiluminescent HRP Substrate; Millipore, Billerica, MA).  All antibodies were purchased from Santa Cruz Biotechnology, Inc., Santa Cruz, CA.

***Western for UCP-1:*** Approximately 30 µg of protein from the IBAT was separated by 10% SDS-PAGE. The gel was transblotted onto a PVDF membrane and dried overnight. After drying, proteins were detected on the PVDF membrane using Sypro Ruby Protein Blot Stain (Molecular Probes, Inc., Eugene, OR) and exposed to ultraviolet light using AlphaEase FC (Fluor Chem 8800) software and a MultiImage Light Cabinet (Alpha Innotech Corporation, San Leandro, CA).Then the membrane was blocked with non-fat milk powder washed with in Tris-buffered saline and probed for UCP-1 (1:1000 rabbit polyclonal primary antibody procured from Abcam, Cambridge, MA, then 1:3000 goat anti-rabbit (GAR) AP detection from BioRad Laboratories, Hercules, CA). Signals were detected using a chemiluminescence kit (Amersham ECL kit). This method was a modification of the method used by Petrovic, N.

***RT-QPCR***

RNA was isolated from EWAT and IBAT fat pads with the RNeasy Lipid Tissue mini kit (Qiagen, Valencia, CA) including a DNAse step. One microgram of total RNA was reverse transcribed to cDNA using QIAGEN reverse transcriptase following manufacturer’s instructions (QuantiTect Reverse Transcription, Qiagen, Courtaboeuf, FRANCE). mRNA expression using real time PCR was performed as described previously . The relative level of expression between control (USO) and treated and pair fed groups (HSO and PSO) was calculated using the ΔΔ*Ct* formula.

**References**

[1] Ollero, M, Junaidi, O, Zaman, MM, et al., Decreased expression of peroxisome proliferator activated receptor gamma in cftr-/- mice, J Cell Physiol, 2004;200:235-244.

[2] Petrovic, N, Shabalina, IG, Timmons, JA, et al., Thermogenically competent nonadrenergic recruitment in brown preadipocytes by a PPARgamma agonist, Am J Physiol Endocrinol Metab, 2008;295:E287-296.

[3] Gora, S, Maouche, S, Atout, R, et al., Phospholipolyzed LDL induces an inflammatory response in endothelial cells through endoplasmic reticulum stress signaling, FASEB J, 2010;24:3284-3297.
