# Supplementary material for: A diet containing soybean oil heated for three hours increases adipose tissue weight but decreases body weight in C57BL/6 J mice
Source: Lipids Health Dis. 2013 Mar 6;12:26. doi: 10.1186/1476-511X-12-26 (PMC3599973; doi:10.1186/1476-511X-12-26)
Supplement: Additional file 2: Table S2 — Composition of the HSOa and USOb diets. [file 1476-511X-12-26-S2.doc]

**Supplementary Table**

**Table2: Composition of the HSO^a^ and USO^b^ diets**

| Ingredients | Low fat diet with heated soybean oil (HSO) | Low fat diet with un-heated soybean oil (USO) |
| --- | --- | --- |
| Casein, 80mesh, g/kg | 190 | 190 |
| L-Cystine, g/kg | 2.8 | 2.8 |
| Corn Starch, g/kg | 298 | 298 |
| Maltodextrin 10, g/kg | 33.2 | 33.2 |
| Sucrose, g/kg | 332 | 332 |
| Cellulose, BW200, g/kg | 47.4 | 47.4 |
| Soybean oil, heated, g/kg (% of kcal) | 14.2 (3.15) | 0 (0) |
| Soybean oil, unheated, g/kg (% of kcal) | 0 (0) | 14.2 (3.15) |
| Lard, g/kg (% of kcal) | 28.4 (6.3) | 28.4 (6.3) |
| Mineral mix , g/kg | 9.5 | 9.5 |
| Dicalcium Phosphate | 12.3 | 12.3 |
| Calcium Carbonate | 5.2 | 5.2 |
| Potassium Cirtate | 15.6 | 15.6 |
| Vitamin mix, g/kg | 9.5 | 9.5 |
| Choline Bitartrate, g/kg | 1.9 | 1.9 |
| PV value (mean±SEM) | 5.81 meq/kg | 1.63 meq/kg |
| Conjugated Dienes, mmol/Kg (mean±SEM) | 793 ± 5.69 | 380±2.55 |
| Malondialdehyde, mmol/Kg (mean±SEM) | 0.014±0.0004 | 0.014±0.0006 |

^a^HSO=Heated Soybean Oil; ^b^USO=Unheated Soybean Oil.
