# Supplementary material for: A diet containing soybean oil heated for three hours increases adipose tissue weight but decreases body weight in C57BL/6 J mice
Source: Lipids Health Dis. 2013 Mar 6;12:26. doi: 10.1186/1476-511X-12-26 (PMC3599973; doi:10.1186/1476-511X-12-26)
Supplement: Additional file 3: Figure S2 — Mean body weights of mice over the 16 week study period. [file 1476-511X-12-26-S3.docx]

Additional file 3-Figure S2

Mean body weights of mice over the 16 week study period.
